# Practice Induces Function-Specific Changes in Brain Activity

**DOI:** 10.1371/journal.pone.0003270

**Published:** 2008-10-01

**Authors:** Tamar R. van Raalten, Nick F. Ramsey, Jeff Duyn, Johan M. Jansma

**Affiliations:** 1 Rudolf Magnus Institute of Neuroscience, Department of Neurology and Neurosurgery, University Medical Centre Utrecht, Utrecht, The Netherlands; 2 Rudolf Magnus Institute of Neuroscience, Department of Child and Adolescent Psychiatry, University Medical Centre Utrecht, Utrecht, The Netherlands; 3 Advanced MRI, Laboratory of Molecular Functional Imaging, National Institute of Neurological Disorders and Stroke, National Institutes of Health, Bethesda, Maryland, United States of America; 4 National Institutes of Health, National Institute of Mental Health, Section on Neuroimaging in Mood and Anxiety Disorders, Bethesda, Maryland, United States of America; Victoria University of Wellington, New Zealand

## Abstract

**Background:**

Practice can have a profound effect on performance and brain activity, especially if a task can be automated. Tasks that allow for automatization typically involve repeated encoding of information that is paired with a constant response. Much remains unknown about the effects of practice on encoding and response selection in an automated task.

**Methodology:**

To investigate function-specific effects of automatization we employed a variant of a Sternberg task with optimized separation of activity associated with encoding and response selection by means of m-sequences. This optimized randomized event-related design allows for model free measurement of BOLD signals over the course of practice. Brain activity was measured at six consecutive runs of practice and compared to brain activity in a novel task.

**Principal Findings:**

Prompt reductions were found in the entire cortical network involved in encoding after a single run of practice. Changes in the network associated with response selection were less robust and were present only after the third run of practice.

**Conclusions/Significance:**

This study shows that automatization causes heterogeneous decreases in brain activity across functional regions that do not strictly track performance improvement. This suggests that cognitive performance is supported by a dynamic allocation of multiple resources in a distributed network. Our findings may bear importance in understanding the role of automatization in complex cognitive performance, as increased encoding efficiency in early stages of practice possibly increases the capacity to otherwise interfering information.

## Introduction

Practice can have a profound effect on performance and underlying brain activity especially if a task can be automated. Tasks that allow for automatization typically involve repeated encoding of information that is paired with a constant response [1]. While previous studies have demonstrated the profound effects of automatization on working memory [2]–[4], much remains unknown about how automatization affects function-specific effects related to encoding and response selection in an automated task.

Decreases in working memory activity after practice have been reported in a wide range of cognitive tasks; such as verb generation [5], mirror reading [6], [7], delayed response tasks [4], [8] and motor sequence learning [9], [10] and have been interpreted in terms of reduced demands on domain-general cognitive control resources that support early learning or novel task performance [2], [11]. It has also been shown that practice-induced activity decreases are closely related to one's capacity to concurrently perform an additional cognitive task [3]. Better understanding of the mechanism behind automatization may explain how automatization can contribute to complex cognitive performance such as dual tasking.

To investigate function-specific effects of automatization we build upon our previous work in which we examined automatization by means of a Sternberg Task [12]. Performance of this task involves an encoding phase during which information is presented that is briefly memorized, and a response phase including a probe stimulus that requires a decision whether it matches the previously presented information or not. The blocked design we employed in our previous studies [3], [4] did not allow investigation of function-specific changes in brain activity associated with encoding and response selection. In addition, it was not possible to assess changes in brain activity over the course of practice. To investigate function-specific changes in brain activity as a result of practice we used a pseudo-random event-related design in which encoding and response phases in a Sternberg task were controlled by means of m-sequences [13]. This is a novel method that allows for model-free measurement of BOLD signals and optimal separation of BOLD signals of rapidly displayed stimuli. Brain activity was measured at six consecutive runs of practice to measure changes in activity during encoding and response selection over the course of practice. Based on current theories of practice we hypothesize that the course of activity decreases in brain areas associated with working memory function is the same for encoding and response selection, while both functions may show independent courses in activity changes in function-specific networks.

## Methods

### Participants

Eleven right-handed subjects (M/F 6/5, mean age 33.0 (±2.9)) participated in the study. Before the functional MRI (fMRI) session, all subjects gave written informed consent to participate in the study, which was approved by the Intramural Review Board (IRB) of the National Institute of Neurological Disorders and Stroke at the National Institutes of Health under protocol #00-N-0082. Participants were provided with earplugs to protect their hearing from the acoustic noise generated by the MRI gradient system.

### Task

**Figure 1:**
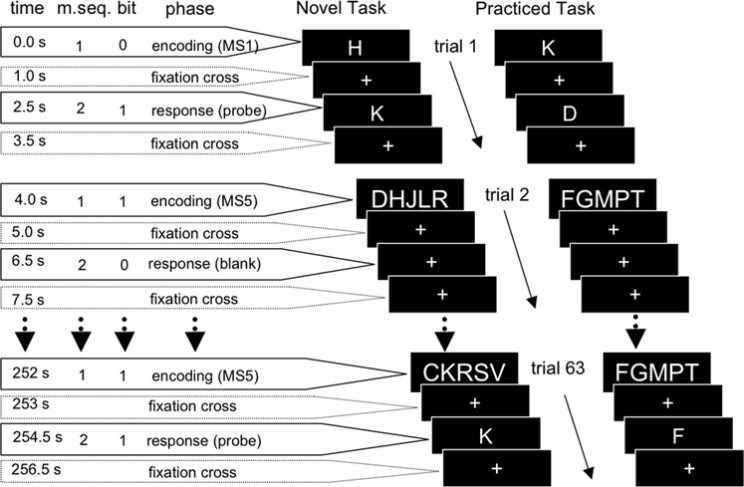
Cognitive Paradigm. The timeline is shown for the cognitive experiment. Two m-sequences (m.seq.) of 63 bits control the encoding phase (1) and the response phase (2). Each trial starts with the encoding phase followed by a brief delay and the response phase. Where bits are 0 (baseline); memory sets with 1 letter (MS1) are presented during the encoding phase and blank trials are presented during the response phase. Where bits are 1; 5-letter memory sets (MS5) are presented during the encoding phase and a probe stimulus during the response phase. In the novel task the letters presented during the encoding phase were different in each MS1 and MS5 trial. In the practiced task, the same five letters were repeated in each MS5 trial.

We based the task used in our study on a Sternberg task-paradigm [12] (figure 1). This task has been used extensively in fMRI studies and it has been shown to reliably activate regions associated with working memory [4], [14]–[22]. It allows for trial-by-trial measurement of the level of performance (reaction time (RT) and error rate) to verify that subjects are executing the task as required.

In our experiment participants were instructed to memorize either one or five letters that were visually presented (memory set). To increase similarity between stimuli, all of the letters used in the task were consonants. The memory set was followed by presentation of a probe stimulus. Participants were instructed to decide as fast as possible whether the probe belonged to the memory-set (target) or not (non-target).

Tasks were presented in eight runs of approximately five minutes with each run containing 68 trials of 4000 ms duration. Each trial started with an encoding phase during which the memory set was presented for 1500 ms. This was followed by a delay period of 1500 ms in which a fixation cross was displayed. The brief delay was followed by the response phase, which involved the presentation of a probe stimulus for 500 ms followed by another fixation-cross for 500 ms (figure 1).

In the first two runs, the memory sets for each trial were randomly generated out of ten consonants. Because memory sets were novel in each trial these runs are denoted “novel task” (NT1 and NT2). In the following six runs all trials used the same fixed memory set. These runs are denoted “practiced task” (PT1–PT6). The constant stimulus-response associations in PT are thus practiced in six runs allowing automatization to be established over time. The stimuli in PT were chosen from a different set of consonants than NT to prevent interference.

The first NT run was used to select regions of interest (ROI) representing brain areas involved with encoding and response phases. The second NT run was used to establish signal level for NT performance in the ROI's, which was used as reference for comparison of activity during PT.

### The M-sequence

A 63 element binary m-sequence consisting of 32 positive and 31 negative bits was used to control the timing of the presentation of the task stimuli [13]. The primary sequence was used to control the encoding phase. Each bit of the sequence belonged to one trial. If the sequence was negative then a one-letter memory set was presented (baseline condition for encoding phase). If the sequence was positive a five-letter memory set was presented. The sequence was shifted nine bits to create an independent but related sequence to control the response phase. If this sequence was negative no stimulus was presented (baseline condition for the response phase). If this sequence was positive a probe letter was presented. The m-sequence was extended by inserting a replica of the last five bits at the beginning of the sequence to allow removal of the initial BOLD transient, yielding an extended sequence of 68 bits. The uneven runs used the primary versions of the sequence, while the even runs used an inverted version (positive and negative bits switched).

### Functional MRI

Data was acquired on a 3T GE MR system. Image signal-to-noise ratio (SNR) was boosted by employing multi-channel MRI with a custom-built helmet-type 16- channel receive array that fits tightly around the head [23], connected to a custom-built 16-channel MRI receiver [24]. A single-shot rate-2 sensitivity-encoded (SENSE) [25] echo-planar imaging (EPI) [27] was employed for fMRI acquisition. The EPI matrix size was 96 by 72, and the field of view (FOV) 224 mm2, leading to a nominal in plane resolution of 2.3 mm2. Slice thickness was 2.0 mm, with a slice gap of 0.3. Echo time (TE) was 32 ms, repetition time (TR) was 2000 ms, and flip angle 90 degrees. Tasks were presented in three runs of 290 functional scans with approximately one-minute period in between. A video projector presented stimuli on a small screen attached to the head-coil in the scanner. Participants could see the screen via a mirror also attached to the head-coil. Subjects were instructed to respond to each probe as quickly as possible by ion of the pushing a button with the index finger of the right hand to targets or with the middle finger of the right hand to non-targets.

### Data preprocessing and statistical analysis

All fMRI data were analyzed off-line on a multimode Linux/PC reconstruction cluster using IDL™. Image reconstruction was performed as described previously and included direct Fourier transform of the ramp-sampled data, EPI ghost correction using a navigator echo [26] and SENSE unfolding as well as image intensity correction based on coil sensitivity reference maps derived from the array data itself [27].

First and second order trends were removed from the fMRI signal per voxel. After this, an outlier test was performed, which removed all time points larger than three standard deviations away from the mean. Trend correction for first and second order was repeated after outlier correction. The input function (primary m-sequence) was balanced to have a mean of zero. Because there were two scans per trial, the sequence was interleaved with zeros in order to have a sequence length equal to the number of scans. Analysis of brain activation was performed by calculating the cross-covariance of this input function with the image intensity on a voxel by voxel basis, for all 63 temporal shifts [13] by multiplication in Fourier domain. Covariance values were transformed into t-values by dividing each value by an estimate of the temporal noise level. The temporal noise value in the fMRI signal was estimated by calculating the temporal standard deviation in covariance values over shifts 20 to 63, where no covariance peaks related to our experimental paradigm were present. Subsequently, the correlation maps for ten shifts (or a 20 s period) following the expected correlation peak were spatially normalized to the MNI305 standard brain, as it was expected that the BOLD curve would be fully covered by this segment. These maps were transformed into group activity maps by testing the value in each voxel against zero over all subjects. Two covariance peaks were expected: The first peak related to the encoding phase with an onset at shift zero, the second peak related to the response phase with an onset at shift nine.

Regions of interest (ROI) for encoding phase and response phase were created by combining neighboring voxels that reached a threshold of t>3.71 (p<0.0001 uncorrected) in the group map of the NT1, at shift 2 and at shift 11 (corresponding to the fMRI signal at 4000ms after PS presentation). These signals were analyzed using multivariate analysis (repeated measurements (executed with SPSS ™ 11.0)).

## Results

### Performance

**Figure 2:**
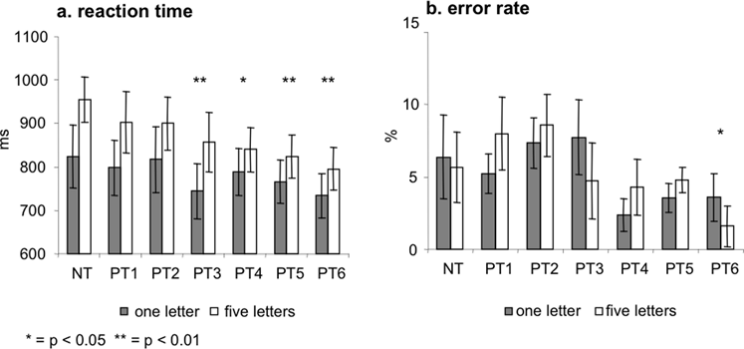
Performance. a. reaction time in milliseconds (left) and b. % error rate (right) for trials with one-letter memory sets and five-letter memory sets. Performance measures are displayed for novel task (NT) and each practice run (PT1-PT6)

We examined the behavioral effect of practice for changes in performance over all practice runs and by comparison of each practice run with NT2. Overall task performance was averaged over responses in one-letter memory set (MS1) and five-letter memory set (MS5) trials and for the difference in performance between responses in MS1 trials and responses in MS5 trials. For reaction time (RT) there was a significant performance improvement over all runs (F = 5.40, p<0.01). Practice runs three through six showed a significant improvement compared to the novel task (see table 1 and figure 2). There was also a significant overall improvement in error rate (F = 2.91, p<0.05) and a significant improvement in the sixth run of practice compared to NT (F = 5.91, p<0.04). The differences between MS5 and MS1 in RT and error rate were not significantly changed by practice (see table 1 and figure 2).

### Overview of regions of interest

**Figure 3:**
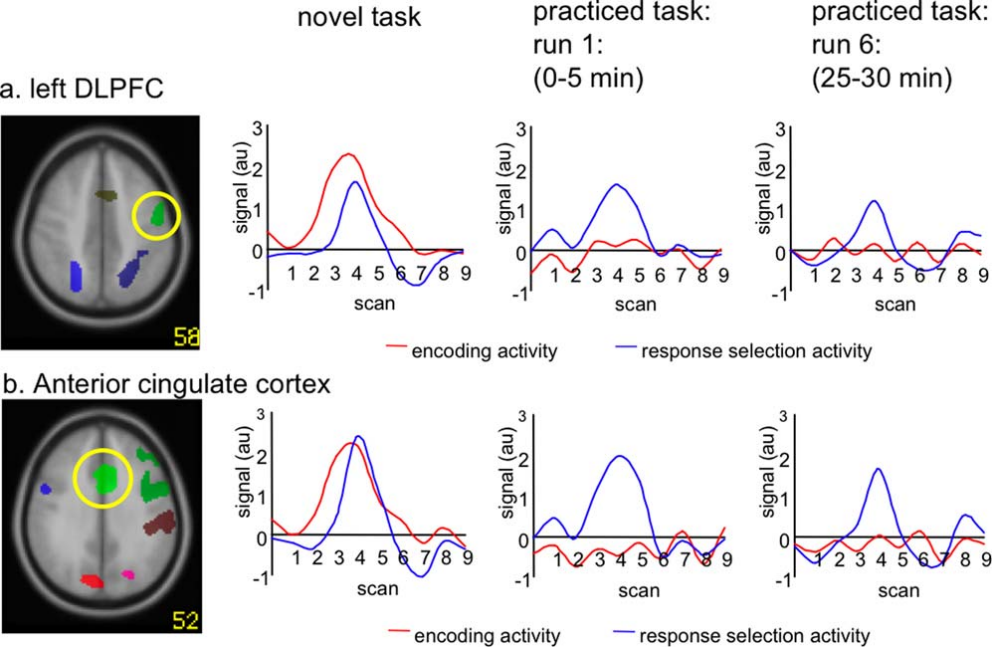
Heterogeneous effect of practice on regions activated by both encoding and response selection. Example of bold activity (arbitrary units) in regions activated by both phases: a. left DLPFC (top) and b. anterior cingulate cortex (bottom) during the novel task (left), after one practice run (middle) and six practice runs (right); showing the heterogeneous effects of practice for encoding and response selection.

**Figure 4:**
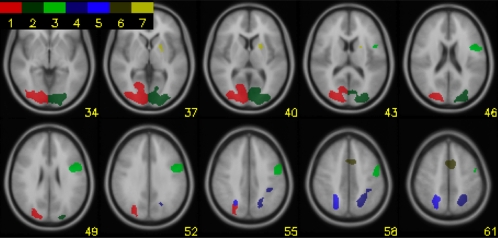
Encoding ROI's. ROIs showing activity related to encoding. The numbers in the color bar refer to the encoding phase ROIs (E1–E7) in table 2.

**Figure 5:**
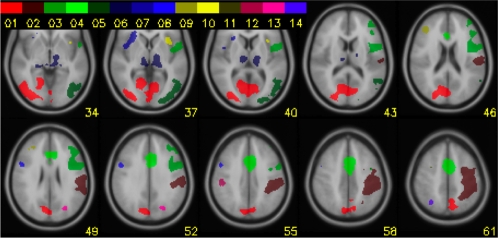
Response Selection ROI's. ROIs showing activity related to the response selection. The numbers in the color bar refer to the response selection ROIs (RS1–RS14) in table 3.

Encoding and response selection activated distinct cortical networks with limited overlap (see figure 3). Encoding ROIs are described in table 2 and figure 4. Response selection ROIs are listed in table 3 and figure 5.

**Table 1:** Practice and Performance

a.	overall		difference	
reaction time	(MS1 and MS5)		(MS1 vs MS5)	
contrast	F(1,10)	p	F(1,10)	p
multivariate	5.40*	**<0.01**	1.67**	0.17
NT-PT1	3.02	0.11	0.06	0.46
NT-PT2	3.16	0.11	2.33	0.16
NT-PT3	**17.5**	**<0.01**	0.25	0.63
NT-PT4	**6.37**	**0.03**	4.68	0.06
NT-PT5	**11.6**	**<0.01**	2.39	0.15
NT-PT6	**15.7**	**<0.01**	4.16	0.07
* df = (2.8,28.4) ** df = (4.3, 43.2) (Huynh-Feldt corrected)				

b.	overall		difference	
error rate	(MS1 and MS5)		(MS1 vs MS5)	
contrast	F(1,10)	p	F(1,10)	p
multivariate	2.91*	**0.05**	0.008**	0.93
NT-PT1	0.08	0.78	2.67	0.14
NT-PT2	1.23	0.30	0.79	0.40
NT-PT3	0.01	0.94	0.41	0.54
NT-PT4	3.10	0.11	1.59	0.24
NT-PT5	1.23	0.30	0.82	0.39
NT-PT6	**5.91**	**0.04**	0.09	0.77
* df = (2.8,28.4) ** df = (4.3, 43.2) (Huynh-Feldt corrected)				

a. reaction time (top) and b. error rate (bottom). Measures were tested over all runs (1st row) and between novel task (NT) and each practice run (PT), across one-letter (MS1) and five-letter (MS5) memory set trials (1st column) and for MS1 trials vs. MS5 trials (2nd column). Significant results are displayed in bold.

**Table 2:** Encoding ROI's

ROI	Region	abbr.	BA	NV	x	y	z	tmax
E1	right calcarine sulcus	Rcalc	18	2886	14	−94	2	12.87
E2	left calcarine sulcus	Lcalc	18	2334	−12	−94	−2	12.75
E3	dorsolateral prefrontal cortex	Ldlpfc	9	1044	−56	−2	42	6.94
E4	left superior parietal cortex	Lspc	7	693	−24	−58	46	6.46
E5	right superior parietal cortex	Rspc	7	475	24	−50	46	4.06
E6	Anterior cingulate cortex	SMA	24	472	−4	2	58	8.36
E7	left putamen	Lput	Na	112	−22	2	−2	3.53

Description of ROIs showing activity correlated with encoding phase. (Abbreviations: E = encoding; BA = Brodmann Area; NV = number of voxels in ROI (size of ROI); x, y, z = MNI coordinates of voxel with highest t-value in ROI; tmax: maximum t-value in ROI).

**Table 3:** Response Selection ROI's

ROI	Region	abbr.	BA	NV	x	y	z	tmax
RS1	right occipital cortex	Rocc	18/19	4054	18	−64	8	7.42
RS2	left primary sensorimotor cortex	Lpsmc	4	3723	−38	−22	54	11.84
RS3	left dorsolateral prefrontal cortex	Ldlpfc	9/46	2527	−52	2	14	8.05
RS4	anterior cingulate cortex	ACC	32	2105	−4	−4	56	10.11
RS5	left occipital cortex	Locc	19	1457	−52	−68	6	6.8
RS6	Thalamus	thal	Na	874	−14	−26	2	6.21
RS7	right operculum	Roper	45	348	30	20	0	5.03
RS8	right dorsolateral prefrontal cortex	Rldpfc	46	152	52	2	38	5.49
RS9	right ventrolateral prefrontal cortex	Rvpfc	47	149	40	30	20	3.94
RS10	left operculum	Loper	45	132	−40	12	4	5.54
RS11	right precentral gyrus	Rpcg	6	99	28	−2	54	6.48
RS12	right postcentral gyrus	Rpocg	2	96	54	−24	40	5.85
RS13	left cuneus	Lcun	19	95	−24	−76	30	6.06
RS14	right superior parietal cortex	Rspc	7	81	30	−58	48	4.13

Description of ROIs showing activity correlated with response phase. Abbreviations: RS = response selection; BA = Brodmann Area; NV = number of voxels in ROI (size of ROI); x, y, z = MNI coordinates of voxel with highest t-value in ROI; tmax: maximum t-value in ROI).

#### Encoding

During the encoding phase bilateral regions in the occipital cortex and superior parietal cortex and the dorsal part of the anterior cingulate cortex were activated. In addition, there was activity in the left dorsolateral prefrontal cortex and the putamen.

#### Response selection

During the response phase there was also bilateral activity in the occipital cortex, but closer to the extrastriate and middle occipital gyrus, the DLPFC, the ACC, the operculum and the thalamus. In addition, ROIs were identified in the left primary sensorimotor cortex and the cuneus. In the right hemisphere we identified ROIs in the ventrolateral prefrontal cortex, the postcentral gyrus, the precentral gyrus and the superior parietal cortex.

### Changes in activity related to practice

**Figure 6:**
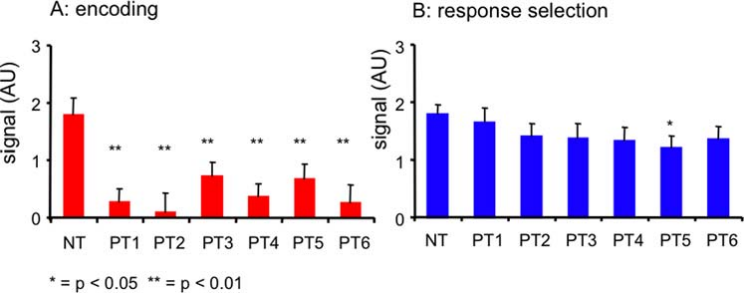
Practice and brain activity. a. activity in arbitrary units during encoding averaged over encoding phase ROIs (left) and b. activity during response selection averaged over response selection ROIs (right). Activity is displayed for novel task (NT) and each practice run (PT1–PT6).

To examine function-specific effects of practice we tested activity averaged over all ROIs in the encoding and response selection networks (table 4 and figure 6) and in each individual ROI of the separate encoding network (table 5) and response selection network (table 6) for changes in activity across all practice runs and between each PT run compared to NT.

**Table 4:** Practice and Brain Activity

	encoding		response selection	
contrast	F(1,10)	p	F(1,10)	p
multivariate	7.96*	**<0.01**	1.19**	0.32
NT-PT1	**31.97**	**<0.01**	0.38	0.55
NT-PT2	**19.67**	**<0.01**	0.30	0.16
NT-PT3	**18.23**	**<0.01**	2.48	0.15
NT-PT4	**18.51**	**<0.01**	3.59	0.09
NT-PT5	**14.24**	**<0.01**	4.97	0.05
NT-PT6	**36.39**	**<0.01**	2.99	0.11
* df = (6,60) ** df = (6,60) Huynh-Feldt corrected				

Tests for significant effects of practice on encoding activity averaged over all encoding ROIs and response selection activity averaged over all response selection ROIs. Signals were tested over all runs (1^st^ row) and between novel task (NT) and each practice run (PT). Significant results are displayed in bold.

**Table 5:** Practice and Encoding Activity

	multi-variate		NT-PT1		NT-PT2		NT-PT3		NT-PT4		NT-PT5		NT-PT6	
	F	p	F	p	F	p	F	p	F	p	F	p	F	p
Rcalc (E1)	**4.93**	**<.01**	**17.0**	**0.00**	**10.6**	**0.01**	**19.5**	**<.01**	**14.6**	**<.01**	**9.56**	**0.01**	**21.3**	**<.01**
Lcalc (E2)	**7.29**	**<.01**	**27.9**	**0.00**	**12.7**	**0.01**	**28.7**	**<.01**	**13.7**	**<.01**	**20.5**	**0.00**	**31.9**	**<.01**
Ldlpfc (E3)	**6.16**	**<.01**	**15.8**	**0.00**	**15.2**	**<.01**	**6.88**	**0.03**	**19.7**	**<.01**	**10.1**	**0.01**	**23.7**	**<.01**
Lspc (E4)	**4.81**	**<.01**	**20.5**	**0.00**	**13.9**	**0.00**	3.86	0.08	**10.7**	**0.01**	**10.4**	**0.01**	**22.9**	**<.01**
Rspc (E5)	**3.62**	**<.01**	**11.0**	**0.01**	**12.5**	**0.01**	3.13	0.11	**6.33**	**0.03**	**11.1**	**0.01**	**12.6**	**0.01**
SMA (E6)	**7.46**	**<.01**	**10.2**	**0.01**	**12.5**	**0.01**	3.48	0.09	**12.7**	**0.01**	**6.61**	**0.03**	**18.8**	**<.01**
Lput (E7)	**3.14**	**0.01**	**6.3**	**0.03**	**10.3**	**0.01**	0.64	0.44	**5.13**	**0.05**	0.96	0.35	**6.7**	**0.03**

Multivariate tests for signals in ROIs related to encoding. Signals were tested over all runs (1st column) and between novel task (NT) and each practice run (PT). Significant results are displayed in bold. (For abbreviations see table 2).

**Table 6:** Practice and Response Selection Activity

	multi-variate		NT-PT1		NT-PT2		NT-PT3		NT-PT4		NT-PT5		NT-PT6	
	F	p	F	p	F	p	F	p	F	p	F	p	F	p
Rocc (RS1)	0.89	0.51	0.28	0.60	2.48	0.15	0.48	0.51	0.19	0.67	0.69	0.42	1.71	0.22
Lpsmc (RS2)	1.37	0.34	0.84	0.38	1.57	0.24	3.69	0.08	3.67	0.09	**7.52**	**0.02**	1.07	0.33
Ldlpfc (RS3)	1.27	0.29	0.49	0.50	1.53	0.24	1.32	0.28	4.60	0.06	**9.60**	**0.01**	**5.60**	**0.04**
ACC (RS4)	1.49	0.20	3.47	0.09	1.14	0.31	3.27	0.10	**5.01**	**0.05**	**6.78**	**0.03**	**7.20**	**0.02**
Locc (RS5)	1.40	0.22	1.11	0.32	3.18	0.11	3.90	0.08	4.61	0.06	**4.83**	**0.05**	1.83	0.21
thal (RS6)	1.59	0.19	1.00	0.34	0.70	0.42	2.84	0.12	0.67	0.43	1.90	0.20	0.11	0.75
Roper (RS7)	0.75	0.61	0.04	0.86	0.08	0.78	0.12	0.74	1.28	0.30	0.75	0.41	0.92	0.36
Rldpfc (RS8)	1.51	0.20	1.73	0.22	2.87	0.12	2.27	0.16	**7.66**	**0.02**	**8.10**	**0.02**	3.33	0.10
Rvpfc (RS9)	0.70	0.63	0.01	0.95	0.47	0.51	0.00	0.99	2.20	0.17	0.96	0.35	1.04	0.33
Loper (RS10)	0.96	0.45	0.01	0.94	0.01	0.91	0.78	0.40	0.90	0.37	**7.06**	**0.02**	0.88	0.37
Rpcg (RS11)	2.32	0.05	2.03	0.18	1.75	0.22	2.56	0.14	**7.77**	**0.02**	**14.59**	**0.01**	**5.06**	**0.05**
Rpocg (RS12)	1.12	0.37	0.07	0.79	2.15	0.17	0.42	0.53	2.16	0.17	3.00	0.11	0.05	0.83
Lcun (RS13)	0.53	0.78	0.01	0.96	0.44	0.52	0.70	0.42	0.30	0.60	3.10	0.10	1.10	0.32
Rspc (RS14)	1.33	0.26	4.72	0.06	0.15	0.71	0.00	1.00	1.58	0.24	0.00	0.99	0.22	0.65

Multivariate tests for ROI signals related to response selection. Signals were tested over all runs (1st column) and between novel task (NT) and each practice run (PT). Significant results are displayed in bold. (For abbreviations see table 3).

#### Effects of practice on encoding activity

The multivariate test for changes in activity (averaged over all encoding ROIs) shows a significant effect of practice across all six practice runs (table 5). Tests for changes in activity compared to the novel task show a significant decrease in all practice runs (p<0.01) (table 5). Separate tests for each ROI show significant decreases in bilateral visual cortex (E1 and E2) and left DLPFC (E3). Bilateral SPC (E4 and E5) and SMA (E6) show significant decreases in activity for all but the third practice run. Left PUT (E7) show a significant decrease for all practice runs, except runs three and five (table 5).

#### Effects of practice on response selection activity

The multivariate test for changes in activity (averaged over all response selection ROIs) across all practice runs was not significant (table 6). In addition, there was no significant change in activity from the novel task at any practice run (table 6). In tests of separate ROIs (table 6), we found a significant decrease in signal compared to the novel task in the lPSMC (RS2) in practice run 5, in the lDLPFC (RS3) in runs 5 and 6, in the ACC (RS4) in runs 4, 5 and 6, in lOCC (RS5) in practice run 5, rDLPFC (RS8) in practice run 4 and 5, in lOPER (RS10) in practice run 5, and in rPCG (RS11) in practice run 4, 5 and 6.

In summary, practice reduced activity in function-specific regions associated with encoding and response selection. However, signal in encoding areas was reduced in all regions of the network after the first practice run, while in the response selection areas practice decreased activity only after the third practice run, and only in a subset of regions.

#### Heterogeneous effect of practice on encoding and response selection activity

Figure 3 illustrates the distinct effect that practice has on the encoding and response phase activity by showing the complete BOLD curves for left DLPFC and ACC. During the encoding phase, BOLD activity was practically absent in the first runs of practice in both regions (3a, 3b; red lines). For the response phase, BOLD activity is still visually detectable in lDLPFC (3a, blue line), and ACC (3b, blue line), up to the last run of practice.

## Discussion

### Summary

This study examined the effect of practice on brain activity associated with encoding and response selection. We used an optimized pseudo-random event-related design that isolated effects of practice in the encoding phase and response phase of a Sternberg task, at six runs of practice. Performance and brain activity at each practice run were compared to that of a similar task with novel stimuli. Our behavioral results show that practice gradually but significantly improved performance, confirming automatization of task performance [1]. Practice promptly reduced activity across the entire regional network involved in encoding at the first run of practice, before response selection activity and performance were affected. Changes in response selection activity emerged at the third run of practice and were not present in all regions, but specific for ACC, left and right DLPFC, lPSMC, lOCC, rPCG and lOPER. Our results indicate that automatization can induce independent changes in function-specific brain regions over the course of practice.

### Heterogeneous effects of practice on encoding and response selection

In the novel task, encoding and response selection activated regions associated with working memory in left DLPFC, and SMA/ACC and right superior parietal cortex. This common activation of the working memory network during different phases of novel Sternberg performance supports the notion of a scaffolding system that contributes to novel task performance [2]. However, practice induced different courses of activity reductions in working memory activity for the encoding and the response selection. Practice immediately reduced activity in the encoding network at the first run of practice in left DLPFC and ACC. In sharp contrast, response selection activity in these regions did not show any effect of practice over the course of three runs with repeated memory sets. This indicates that DLPFC and ACC were activated to an extent specifically needed for each phase at the different runs of practice. This divergent pattern of activity changes for encoding and response selection does not support the notion that domain-general control resources are reduced as the need to “scaffold” task performance decreases with practice [2]. Consequently, these findings do not seem to support our hypothesis based on this idea. Our findings are more in line with the idea of decentralized theories of working memory function [28]–[30]. From this perspective practice may independently reduce working memory contributions to different phases of cognitive performance depending on the level of control necessary for each phase. The immediate reductions in activity associated with encoding possibly indicate that practice promptly reduces the need for working memory to support the transformation of visually presented stimuli into a neural representation that facilitates temporary storage of information during the delay [31]–[35]. In addition, the current data shows that a similar amount of practice can only marginally reduce working memory contributions to the response selection phase. This suggests that early in practice, working memory remains engaged to guide response selection based on earlier presented information [15], [34].

The ability to automate task performance has been shown to be important for complex cognitive performance such as the capacity to perform multiple tasks at once [1], [3], [36], [37]. Our results suggest that early in practice reduced demands on encoding may increase one's capacity to process otherwise interfering information. However, performance of an additional task also deteriorates automated performance to some extent [3], [37]. Our results indicate that this could be induced by conflicts at the level of response selection.

### Automatization vs. other effects of practice on brain activity

Our findings are similar to other studies that have reported reductions in brain activity as a result of practice, representing increased efficiency of information processing [38], [39]. However it should be noted that practice-induced activity changes in those studies were either not accompanied with improved performance [39], or selectively involved response selection [38]. Differences with our design are the type of stimuli used [39], and more importantly that stimulus-response associations in those studies changed over trials, which makes it difficult to compare with our findings. Neuroimaging findings of practice effects on brain activity have been inconsistent across studies [40]. The different effects of practice on brain function have been interpreted in terms of reorganization vs. redistribution of activity [40], changes in skill or strategy underlying task performance [41], item-specific or task-skill effects [42], or improved task proficiency [22], [43], [44]. We propose an alternative but important distinction between tasks that allow for automatization and those that cannot be automated, because stimulus-response associations continuously change over the course of practice. Here we show that automatization predominantly affects encoding early in practice even before performance improves.

### Independent encoding and response phase networks

Our finding of distinct networks activated by encoding and response selection is in line with previous studies. The encoding phase in our study activated bilateral SPC. Many other studies have found this region to be activated by visual perception of stimuli in verbal working memory tasks [19], [21], [45]–[47]. It has been postulated that this region is important for encoding and temporary maintenance of information [48]. Response selection activated parts of the prefrontal-striatal-thalamic circuitry (thalamus, left VLPFC and right DLPFC) [49] that is involved in motor response modulation. These regions have been reported to be active during the response phase in delayed response tasks before [15], [45].

We have designated the cognitive functions that we examined encoding and response selection, to emphasize the difference between the functions present in encoding and response phases of cognitive performance that can be automated. Naturally both task phases include many different processes. The encoding phase requires visual perception, encoding and short-term maintenance of the presented stimuli. The response phase also involves visual perception and encoding as well as response selection and execution. Based on the current design it is not possible to distinguish any of these processes within the current results, but we feel that the terms used, describe the most important function associated with the phase. We have restricted our analyses of brain activity to the encoding phase and response phase, while other studies also included the delay [15], [19], [21], [47]. We decided not to separate the encoding phase from the delay, as it is difficult to separate these phases other than to vary the length of the delay period, which is not possible in an m-sequence design. Notably, current emerging views are that these phases activate the same brain systems [48]

### Limitations

Due to limitations in the design practice trials with five-letter memory sets were interleaved with novel one-letter memory sets. This may have prevented continuous rehearsal of the practiced memory set and consequently slowed down the effect of practice on brain function. The period of practice in our study may therefore have not been sufficient to establish a potential relationship between activity and performance changes reported in other studies [22], [38]. Reaction times on baseline trials (one-letter memory set) showed some improvement with practice. Although this may indicate that task performance became more proficient over time (i.e, regardless of whether stimuli were novel or practiced) it does not affect the main conclusion. The design used in our study yields different baselines for encoding and response selection activity. Encoding activity was based on the contrast between five-letter and one-letter memory sets, while response selection activity was derived from the average of all correct responses in one-letter and five-letter trials. This may have affected the level of activity for the different phases. The m-sequence analysis also provides an interaction activity map [13]. This map did not show any significant activity indicating that interaction effects of the encoding and response phase were small.

### Conclusion

This study demonstrates that practice in a visually delivered cognitive task predominantly increases efficiency of encoding in primary visual, prefrontal and parietal cortex. Changes in the cortical network related to response selection as well as performance improvement occur at a later state of practice. Our results indicate that automatization causes decreases in brain activity that are heterogeneous across functional regions and do not strictly track performance improvement. This suggests that cognitive performance is supported by a dynamic allocation of multiple resources in a distributed network. Our findings may further bear importance in understanding the role of automatization in complex cognitive performance, as increased encoding efficiency in early stages of practice possibly increases the capacity to otherwise interfering information.
